# The Effects of Transcranial Direct Current Stimulation (tDCS) in HIV Patients—A Review

**DOI:** 10.3390/jcm13113288

**Published:** 2024-06-03

**Authors:** James Chmiel, Donata Kurpas, Filip Rybakowski, Jerzy Leszek

**Affiliations:** 1 Institute of Neurofeedback and tDCS Poland, 70-393 Szczecin, Poland; 2 Department of Family and Pediatric Nursing, Faculty of Health Sciences, Wrocław Medical University, 51-618 Wrocław, Poland; 3 Department and Clinic of Psychiatry, Poznan University of Medical Sciences, 61-701 Poznań, Poland; 4 Department and Clinic of Psychiatry, Wrocław Medical University, 54-235 Wrocław, Poland

**Keywords:** tDCS, transcranial direct current stimulation, HIV, non-invasive brain stimulation, neurostimulation, neuromodulation

## Abstract

**Introduction**: HIV is a severe and incurable disease that has a devastating impact worldwide. It affects the immune system and negatively affects the nervous system, leading to various cognitive and behavioral problems. Scientists are actively exploring different therapeutic approaches to combat these issues. One promising method is transcranial direct current stimulation (tDCS), a non-invasive technique that stimulates the brain. **Methods**: This review aims to examine how tDCS can help HIV patients. Searches were conducted in the Pubmed/Medline, Research Gate, and Cochrane databases. **Results**: The literature search resulted in six articles focusing on the effects of tDCS on cognitive and behavioral measures in people with HIV. In some cases, tDCS showed positive improvements in the measures assessed, improving executive functions, depression, attention, reaction time, psychomotor speed, speed of processing, verbal learning and memory, and cognitive functioning. Furthermore, the stimulation was safe with no severe side effects. However, the included studies were of low quality, had small sample sizes, and did not use any relevant biomarkers that would help to understand the mechanisms of action of tDCS in HIV. **Conclusions**: tDCS may help patients with HIV; however, due to the limited number of studies and the diversity of protocols used, caution should be exercised when recommending this treatment option in clinical settings. More high-quality research, preferably involving neurophysiological and neuroimaging measurements, is necessary to better understand how tDCS works in individuals with HIV.

## 1. Introduction

The human immunodeficiency virus (HIV) is a chronic disease for which curative treatment is not currently available. According to the World Health Organization (WHO), as of the end of 2021, 39 million people were living with HIV [1]. This disease is a significant global public health issue, having caused over 630 thousand deaths in 2022 [1]. The introduction of antiretroviral therapy in 1996 has helped to reduce the severity of the HIV epidemic, improve survival rates, and enhance the quality of life for patients. However, HIV-related neurocognitive disorders (HANDs) pose significant challenges in managing the disease [2]. Studies have shown that antiviral therapy does not improve cognitive function impairment, and individuals with stable HIV infection still show detectable inflammatory markers associated with cognitive decline [3,4]. HANDs are standard among people with HIV, with at least mild neurological symptoms observed in 30% of asymptomatic HIV cases and 50% of AIDS cases [2,5], and the severity of these disorders varies depending on the stage of infection [6]. HIV significantly affects the brain’s structure and function. It compromises the blood–brain barrier, enabling inflammatory cytokines and neurotoxins to enter the brain via infiltrating macrophages and/or monocytes [7]. While HIV does not directly infect neurons, it targets microglial cells, which can harm synaptodendritic connections and result in neuroinflammation [3,7]. Autopsy studies have shown that the brain is the second most infected organ, following the lungs [8]. HIV impacts various brain regions, disrupting the frontal–striatal–thalamocortical loops and affecting the structure and function of other white matter tracts and nervous systems, such as the parietal and temporal cortex [2,9]. The virus’s neurotoxic effects are most noticeable in the frontal cortex, white matter tracts connecting these regions, and the basal ganglia [2,10]. The described frontal dysfunction may be a consequence of the advanced stage of HIV infection and is secondary to basal ganglia pathology [2,10]. Researchers have found a correlation between frontal neurodegeneration and declines in neurocognitive abilities [2,11].

People with HIV infection often experience cognitive symptoms that affect various aspects of their cognitive abilities. These symptoms include difficulties in episodic memory (remembering past events) [12], prospective memory (remembering to do things in the future) [13], working memory (holding and manipulating information in the mind) [12], semantic memory (knowledge of facts and concepts) [14], attention [12], response inhibition (suppressing inappropriate actions) [15], executive functions (such as planning and problem-solving) [16], visual–spatial skills [17], and speed of information processing [12]. Asymptomatic individuals, on the other hand, tend to have the most significant difficulties with language and verbal functions [16].

Depression is a frequent psychiatric issue that can arise in individuals with HIV, affecting around 40–42% of patients [18]. Unfortunately, it often goes unnoticed and untreated [19]. This is problematic because untreated depression in HIV patients can lead to unfavorable treatment results [20]. There are different approaches to treating depression in this context, including cognitive behavioral therapy [21], which focuses on modifying negative thought patterns and behaviors, as well as the use of antidepressant medications [22].

Given the multiplicity of cognitive deficits in people with HIV and the prevalence of depression, it is an urgent need to develop new non-pharmacological methods to deal with HANDs and depression in HIV. One of them may be transcranial direct current stimulation (tDCS).

tDCS is a neuromodulation technique that has gained attention recently due to its non-invasive nature, cost-effectiveness, safety, and effectiveness. This method uses sponge electrodes soaked in saline to apply a low-intensity current (0.5–2 mA) to the scalp with different polarities (anode and cathode) [23]. The procedure is painless, well tolerated, and generally safe, with minimal side effects [24]. The effects of tDCS vary depending on the current’s polarity [25,26]. Anodal tDCS stimulates the targeted brain region by depolarizing neurons, making them more easily excitable and increasing their spontaneous activity. Cathodal tDCS, on the other hand, inhibits the firing of neurons below the stimulation site by hyperpolarizing them, reducing their excitability and spontaneous activity [27]. If applied for at least three minutes, the neurobiological changes induced by tDCS can last beyond the stimulation period [25]. When the stimulation lasts over 10 min with a current of 1 to 2 mA, these changes can remain stable for at least one hour [28]. A single session of tDCS for up to 15 min can influence cortical excitability for up to 90 min, and this effect can be prolonged with repeated stimulation. The long-term effects of tDCS on cortical excitability are linked to synaptic modulation mechanisms, as shown by studies in humans [29] and animal models [30,31]. TDCS induces changes in synaptic plasticity, especially in glutamatergic synapses, which depend on calcium. Blocking the N-methyl-D-aspartate (NMDA) receptor prevents the secondary effects of anodal and cathodal tDCS, while specific receptor agonists enhance these effects [32,33]. TDCS also reduces GABAergic activity, which may act as a mechanism for gating tDCS-induced plasticity [34]. It modulates the balance of cortical excitation and inhibition by altering the levels of γ-aminobutyric acid (GABA), glutamate/glutamine, and brain-derived neurotrophic factor (BDNF) [35]. Low calcium levels lead to long-term depression (LTD) of the postsynaptic neuron, while high concentrations induce long-term potentiation (LTP) [36,37]. tDCS is widely used to relieve symptoms of many psychiatric and neurological diseases like Parkinson’s disease [38] and ADHD [39] and improve cognitive functions in healthy populations, including working memory [40], episodic memory [41], and executive functions [42].

This review focuses on the uses of tDCS in alleviating the symptoms of HIV. We speculate that tDCS may reorganize damaged brain circuits in HIV and improve aspects of patients’ functioning.

## 2. Methods

### 2.1. Data Sources and Search Strategy

In preparing this review, J. Ch., D. K., F. R., and J. L. conducted an independent online search guided by specific criteria. We employed a set of combined keywords: “transcranial direct current stimulation” or “tDCS” and “HIV”. Our search spanned several databases, including PubMed/Medline, Research Gate, and Cochrane, focusing on publications from January 2008 to February 2024, with the search conducted in February 2024.

### 2.2. Study Selection Criteria

The eligibility criteria for this analysis required that the studies be clinical trials conducted in English and published between 2008 and 2024. These studies had to explore the effects of tDCS on HIV, whether as a primary or secondary outcome. The exclusion criteria ruled out any articles not published in English and all review articles.

### 2.3. Screening Process

Multiple screening processes were implemented to guarantee the inclusion of pertinent research and the rejection of those that did not satisfy the predetermined criteria. Independent reviewers J. Ch., D. K., F. R., and J. L. thoroughly examined the titles and abstracts during the first screening process.

#### 2.3.1. Title and Abstract Screening

To identify studies that met the inclusion criteria, each reviewer independently assessed the titles and abstracts of the available records. The screening criteria at this stage were centered on the impact of transcranial direct current stimulation on HIV and its relevance.

#### 2.3.2. Full-Text Assessment

Following the initial screening of titles and abstracts, the selected papers underwent a detailed full-text review. The reviewers focused on ensuring that the studies were clinical trials conducted in English and published from January 2008 to February 2024, carefully examining each article to confirm that it met the specified eligibility criteria.

## 3. Results

**Figure 1:**
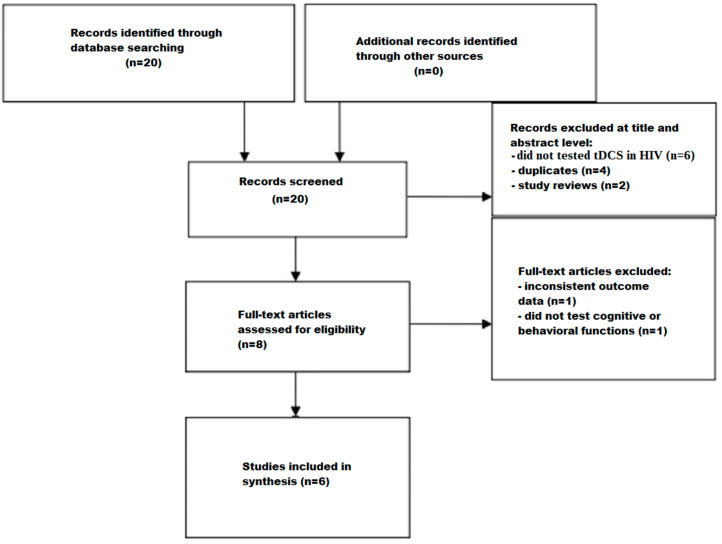
Flow chart depicting the different phases of the systematic review.

The screening process is depicted in a flow chart (Figure 1). Initially, 20 studies were identified using search strategies in various databases. Of these, twelve studies were excluded after reviewing their titles and abstracts; six did not test tDCS in HIV, four were duplicates, and two were study reviews. The remaining eight studies then underwent a comprehensive full-text assessment. Of these, two studies were excluded because one reported inconsistent outcome data and the other did not test cognitive or behavioral functions. After thoroughly reading the texts, six articles were found to meet the criteria for inclusion.

The studies [43,44,45,46,47,48] found were published between 2012 and 2022. Among the included studies, three adopted parallel designs, and one adopted a crossover design. Two studies had one active group. A total of 129 patients were enrolled (active tDCS = 78, sham tDCS = 51). Four of the reviewed studies conducted controlled clinical trials. Random assignment occurred in all controlled clinical trials; all trials used sham stimulation for the control group. In studies [43,44,46], the investigators who conducted the participant assessments were blinded, and in one study [48], the technician who performed the intervention was blinded. A current of 1.5 or 2 mA was used. As for the montage of the electrodes, bipolar montage was the most common. In this configuration, both electrodes were positioned on the brain, and an identical current was passed through the anode and the cathode. In the other studies, a monopolar montage was utilized, where one electrode was placed on the scalp, and the other was positioned extracerebrally, such as on the arm.

### 3.1. Summary of Included Studies

The included studies are summarized in Table 1. In a single-blind randomized study by Ownby and Kim [43], a group of 46 individuals who were being treated for HIV infection and experiencing cognitive difficulties participated. Participants were divided into three groups for this study: (1) cognitive training with active tDCS (16 participants), (2) cognitive training with sham tDCS (15 participants), and (3) watching educational videos with sham tDCS (15 participants). Over a two-week period, they underwent six 20 min training sessions and participated in neuropsychological assessments. In the active tDCS group, the anode was placed over the left dorsolateral prefrontal cortex (DLPFC, located at F3 in the 10–20 System), and the cathode was positioned over the right supraorbital area (FP2). The stimulation was delivered at an intensity of 1.5 mA and lasted for 20 min. Assessments were conducted before the intervention, immediately after the training sessions, and again one month later. The entire intervention concluded within three weeks of the initial baseline assessment. To measure the effects of the intervention on reaction time, participants completed the California Computerized Assessment Package (CCAP). Participants underwent various assessments to evaluate the intervention’s impact on different cognitive and functional areas. Executive functions were assessed using the Stroop Color Word Test, the Iowa Gambling Task, and the Design Fluency subtest from the Delis-Kaplan Executive Function System. Everyday functional abilities were measured with the Medication Management Test—Revised (MMT) and the University of San Diego Scales of Observed Performance (UPSA). The Hopkins Verbal Learning Test—Revised (HVLT-R) was used to assess verbal learning and memory. Attention and working memory were evaluated using the Digit Span subtests from the Wechsler Adult Intelligence Scale, 4th edition (WAIS-IV). The assessment battery also included the Patient’s Assessment of Own Functioning (PAOF), a self-reported measure of cognitive difficulties in areas like language, perception, and memory. Additionally, participants completed the Center for Epidemiological Studies Depression scale (CESD), a self-report tool measuring depressive symptoms.

Ownby and Acevedo [44] conducted a single-blind randomized study to examine the effectiveness and acceptability of cognitive training, both with and without tDCS, in older adults with HIV. The study involved eleven participants (six receiving active tDCS and five receiving sham tDCS), all of whom had HIV-related mild neurocognitive disorder. They completed a series of neuropsychological and self-report assessments, followed by six 20 min cognitive training sessions. During these sessions, participants received anodal tDCS over the left DLPFC at a current of 1.5 mA, with the cathode placed over the right supraorbital area. All sessions were completed within two weeks. The WAIS-IV, which includes the Digit Span subtest, was used to measure working memory and attention, incorporating tests of digit span forward and backward, as well as a number and letter sequencing task. The Trail Making Test Parts A and B assessed executive functions and mental flexibility. The HVLT-R assessed verbal learning and memory. The Grooved Pegboard Test measured psychomotor speed. Depression was calculated using the CESD. The assessment battery also featured the PAOF.

Cody et al. [45] conducted a study with older adults, including 33 individuals with HIV and 33 without HIV. Participants were randomly assigned to undergo speed of processing (SOP) training sessions, receiving either active tDCS or sham tDCS. The study aimed to address two research objectives. The initial goal (Aim 1) of the study was to examine changes in global sleep quality, as assessed by the Pittsburgh Sleep Quality Index (PSQI), and speed of processing (SOP), measured by the Letter and Pattern Comparison Test. This investigation focused on older adults, both with and without HIV, who underwent either actual tDCS or sham tDCS combined with SOP training. The second objective (Aim 2) was to investigate the correlations between changes in global PSQI scores and SOP measures according to the training group. Participants were divided into four groups: (a) HIV-positive with tDCS (n = 17), (b) HIV-positive with sham tDCS (n = 16), (c) HIV-negative with tDCS (n = 17), and (d) HIV-negative with sham tDCS (n = 16). All groups received SOP training plus tDCS consisting of ten one-hour sessions over approximately five weeks. In the active tDCS group, the anodal electrode was positioned over the right inferior frontal cortex near F10, with the cathode placed on the contralateral upper arm. Participants in this group received a 2 mA current for 20 min. Assessments of comprehensive neurocognitive functions were conducted both at baseline and immediately after the test. These included the WAIS Digit Symbol Substitution Test to evaluate visuomotor coordination and attention, the Digit Symbol Copy test to assess psychomotor speed, and the Useful Field of View (UFOV) test to measure visual attention and SOP. For Aim 2, significant correlations were observed between changes in global PSQI scores and SOP measures, specifically with UFOV and Digit Symbol Substitution. Among the HIV-negative participants who received sham tDCS with SOP training, improvements in global PSQI scores were linked to better UFOV performance. Similarly, in the HIV-positive group undergoing sham tDCS with SOP training, enhanced global PSQI scores were associated with improvements in Digit Symbol test performance.

The goal of the study conducted by Fazeli et al. [46] was to investigate the effectiveness of combining SOP cognitive remediation therapy (CRT) with tDCS as a form of neurorehabilitation in older adults living with HIV. A total of 33 HIV-positive adults aged 50 and above participated in the study. They underwent neurocognitive testing and were randomly assigned to either the active tDCS group (n = 17) or the sham tDCS group (n = 16). Both groups completed ten one-hour sessions of SOP CRT over approximately two weeks, with either active or sham tDCS applied for the first 20 min of each session. After the sessions, participants underwent a post-test assessment. The anode (F10 electrode) was placed on the right inferior frontal cortex, and the cathode electrode was positioned on the contralateral upper arm, with stimulation intensity set at 2.0 mA. Cognitive measures included the NIH Toolbox Cognition Battery (NIHTB-CB), which tests executive function (Flanker, Dimensional Card Change Sorting), attention (Flanker), episodic memory (Picture Sequence Memory Test), language (Picture Vocabulary Test, Oral Reading Recognition Test), speed of processing (Letter and Pattern Comparison Test, Digit Symbol Substitution Task), and working memory (List Sorting Test). Additionally, the Useful Field of View Test (UFOV) was administered, comprising subtests for speed of processing (subtest 1), divided attention (subtest 2), selective attention (subtest 3), and a total score (sum of subtests 1–3).

A study conducted by Knotkova et al. [47] aimed to assess the safety, acceptability, feasibility, and clinical outcomes of a two-week tDCS treatment for patients diagnosed with HIV-MDD (HIV-related major depressive disorder). Additionally, the study examined the feasibility of collecting serum and saliva samples to analyze immunity biomarkers. Ten patients participated in the study and underwent baseline evaluation before starting the tDCS treatment. The treatment involved ten daily sessions for two weeks, each lasting 20 min and utilizing a 2 mA current. The anode was positioned over the F3 location (left DLPFC) according to the EEG 10–20 system, and the cathode was placed over the contralateral supraorbital area. Outcome measures were taken at baseline, right after the last tDCS session, and again two weeks following the treatment. Saliva and plasma samples were analyzed using a quantitative microarray to assess TH1/TH2 cytokines. Data from eight subjects were available for analysis. To measure depression, the Hamilton Depression Rating Scale, Montgomery–Asberg Depression Rating Scale (MADRAS), and Mini-Mental State Examination were used.

The objective of the study conducted by Jiang et al. [48] was to investigate the effects of tDCS applied to the dorsal anterior cingulate cortex (dACC) and posterior cingulate cortex (PCC) in individuals living with HIV (PWH). Eleven PWH participated in the study, which consisted of two phases. During Phase 1 of the study, participants were randomly allocated to receive either sham tDCS (4 participants) or cingulate tDCS (7 participants) over a span of 2–3 weeks, totaling ten sessions. Electrode placement was based on the international 10-10 EEG system. To ensure accuracy, the placement of two anodal electrodes at AFz and CPz and two cathodal return electrodes at T7 and T8 was guided by finite element electrical field models. Current intensity was gradually increased to 1.5 mA and applied for 20 min. Additional study visits were arranged to gather neuropsychological and MRI data, beginning with a baseline visit prior to the treatment followed by three follow-up visits—approximately 1 week, 3 weeks, and 3 months after treatment, respectively, labeled as FU1, FU2, and FU3. The type of treatment each participant received was disclosed after FU3. Those in the sham group then entered Phase 2, where they received open-label cingulate tDCS. The study used Wisconsin Card Sorting Test (WCST), Trail Making Test-Part A (TMT-A), TMT-Part B (TMT-B), and the Stroop Color and Word Test (SCWT) to measure executive functions.

### 3.2. Effects of tDCS on Executive Functions

In study [43], there was no significant overall interaction between group and time in the Trail Making Test Part B. However, the comparison between the active and control treatment groups came close to significance at the second follow-up. A significant interaction between group and time was observed in the Stroop test, demonstrating a large effect size. However, this considerable finding was mainly due to a substantial improvement in the group that received cognitive training with sham stimulation. Other interactions showed positive outcomes, although they were not statistically significant. The impact sizes fell within the moderate range.

Study [44] showed an improvement in executive functions measured using the Trail Making Test, Parts A and B.

In study [46], there was an improvement in executive functions, as measured by Dimensional Card Change Sorting and Flanker.

In study [48], tDCS, compared to sham tDCS, reduced Perseverative Errors in the Wisconsin Card Sorting Test but had no impact on Non-Perseverative Errors. It also lowered the ratio score of Trail Making Test-Part B (TMT-B) to Trail Making Test-Part A (TMT-A). However, there was no effect on Stroop Color and Word Test results.

### 3.3. Effects of tDCS on Cognitive Functioning

Study [43] presented varied findings, indicating that CCT with or without tDCS could lead to enhanced cognitive functioning compared to the control group. Regarding PAOF outcomes, analyzing the interaction plots revealed that the average scores for all three groups increased by a comparable degree across the three assessments.

Study [44] reported an improvement in PAOF after tDCS (fewer complaints).

### 3.4. Effects of tDCS on Reaction Time and Psychomotor Speed

In study [43], tDCS did not improve reaction time (CCAP) and psychomotor speed. Although the combined effect of group and time did not yield significant results for the Trails B test, the comparison between the active treatment group and the control group nearly reached significance, indicating a potential trend [t(67.2) = 2.37, *p* = 0.053; d = 0.85]. This difference became significant at the second follow-up [t(67.2) = 3.03, *p* = 0.01; d = 1.09]. The study supported the hypothesis that there could be an improvement in psychomotor speed, as shown by a significant interaction between group and time on the WAIS-IV Coding subtest. However, a closer look at the interaction plot suggested that this effect was primarily driven by the performance of individuals in the CCT with sham tDCS group. The difference between the CCT + sham group and other groups was not statistically significant immediately after the intervention [t(45.6) = 1.02, *p* = 0.57; d = 0.37], but it moved closer to significance at the 1-month follow-up [t(45.6) = 2.21, *p* = 0.08; d = 0.79].

In study [44], results for the Grooved Pegboard dominant hand time showed improvement in the active tDCS group.

In study [45], people with HIV achieved less improvement in the Digit Symbol Copy test after SOP training with active tDCS compared to people after sham tDCS.

### 3.5. Effects of tDCS on Functional Abilities

In study [43], the findings indicate a moderate impact size for the MMT, though it was not statistically significant. The UPSA, on the other hand, showed a negligible effect size.

### 3.6. Effects of tDCS on Verbal Learning and Memory

In study [43], results from the HVLT-R indicated a significant interaction between the treatment group and time, showing differential improvement in both groups. The group that received CCT with active tDCS showed a significant improvement compared to the control group at the immediate follow-up assessment. Although this difference was not significant at the one-month follow-up, it remained substantial.

Study [44] showed improvement in HVLT-R in both groups, but in the active tDCS group, the improvement was greater.

### 3.7. Effects of tDCS on Depression

In study [43], participants generally reported similar levels of depression (CESD) across groups and assessments. However, those in the active treatment group noted an improvement in mood at the 1-month follow-up evaluation.

In study [44], tDCS reduced CESD depression scores.

In study [47], the results showed a significant decrease in depression scores (*p* < 0.0005) after the tDCS treatment. The Hamilton Depression Rating Scale scores for the eight patients averaged 26.3 (s.d. = 5.5) at baseline, 9.9 (s.d. = 4.3) immediately after the last tDCS session, and 7.6 (s.d. = 6.7) at the two-week follow-up. Similarly, the scores on the MADRAS averaged 26.8 (s.d. = 6.7) at baseline, 11.3 (s.d. = 6.9) immediately after the last tDCS session, and 7.0 (s.d. = 7.4) at the two-week follow-up. Mini-Mental State Examination scores remained stable or improved after the treatment.

### 3.8. Effects of tDCS on Working Memory

In study [43], an improvement in working memory was demonstrated in the Digit Span Forward subtest.

In study [44], the findings revealed improvement in working memory, specifically in tasks such as Digit Span Backward and Sequencing.

In study [46], there was no improvement in working memory as measured by the List Sorting Test.

### 3.9. Effects of tDCS on Attention

In study [43], in the Digit Span Forward subtest of attention, the combination of treatment group and time approached statistical significance, showing a substantial effect size. Additionally, the distinction between active tDCS and the control group also nearly reached statistical significance [t(61.3) = 2.19, *p* = 0.08; d = 0.79]. Other subtests designed to measure attention demonstrated positive outcomes, but these interactions were not statistically significant.

The findings of study [44] showed improvement in attention in tasks such as Digit Span Backward and Sequencing.

In study [45], in the Digit Symbol Substitution Test, there was no improvement after active tDCS. All groups improved in the UFOV.

In study [46], no improvement was shown in UFOV subtest 2 (divided attention). The outcomes of subtest 3 (selective attention) improved significantly in the tDCS group at a significance level of *p* < 0.05 after applying correction using Holm’s procedure.

### 3.10. Effects of tDCS on Speed of Processing

In study [45], the HIV-positive group that received tDCS with SOP training showed significant improvements in the Letter Comparison Test. The groups showed no notable variations in their Pattern Comparison or Digit Substitution scores. All groups improved in the UFOV test.

In study [46], an improvement was shown in speed of processing as measured by the Letter and Pattern Comparison Test. The outcomes of the Digit Symbol Substitution Task improved significantly in the tDCS group at a significance level of *p* < 0.05 after applying correction using Holm’s procedure.

### 3.11. Effects of tDCS on Sleep

In study [45], no significant changes were seen in the groups’ overall quality of sleep (all *p* values > 0.05).

### 3.12. Effects of tDCS on Language

In study [46], a language improvement was demonstrated as measured by the Oral Reading Recognition Test. Conversely, there was no evidence of language improvement as measured by the Picture Vocabulary Test.

### 3.13. Effects of tDCS on Episodic Memory

Study [46] found no improvement in episodic memory as measured by the Picture Sequence Memory Test.

### 3.14. Adverse Effects

tDCS was safe, with few side effects. During one tDCS session, two patients experienced a burning sensation, and two others felt an unpleasant tingling or prickling under the electrode. Additionally, individual cases included mild dizziness, restlessness, swollen and painful ankles, muscle spasm in the thigh, worsening of seasonal allergies, gastrointestinal disturbance (diarrhea and nausea), tightness in the chest related to asthma, and pain in the left hip.

## 4. Discussion

### 4.1. General Findings

People with HIV often suffer from cognitive deficits and mental dysfunctions which constitute a significant burden for this group of patients. For this reason, it is necessary to look for methods to help alleviate these deficits. This review examined the potential of transcranial direct current stimulation in people with HIV. Six studies were included. The analyzed manuscripts tested the effects of tDCS on many aspects of functioning, including executive functions, psychomotor speed, attention, verbal learning and memory, depression, working memory, sleep, language, and episodic memory in individuals living with HIV. The studies varied in terms of participants, interventions, and outcome measures. In general, tDCS improved some cognitive functions (executive functions, attention, reaction time, psychomotor speed, speed of processing, verbal learning, and memory), cognitive functioning, functional abilities, and depression indicators. Mixed or null results were obtained in other measurements. Given these outcomes, tDCS appears promising for improving cognitive and functional abilities in people with HIV. Nonetheless, further research is crucial to substantiate these findings and refine treatment protocols. Here, we discuss common findings regarding the positive outcomes that tDCS has demonstrated.

### 4.2. Executive Functions

The studies collectively suggest that tDCS may have a positive impact on executive functions in individuals with HIV. Ownby and Kim [43] demonstrated a significant interaction between group and time for the Stroop test, driven by substantial improvement in the group receiving cognitive training with sham stimulation. Ownby and Acevedo [44] reported improved executive functions measured using the Trail Making Test. Fazeli et al. [46] found improvement in executive functions measured by Dimensional Card Change Sorting and Flanker. In Jiang et al. [48], tDCS decreased Perseverative Errors during the Wisconsin Card Sorting Test (WCST). Different protocols were used—the left DLPFC was stimulated in two studies, the right inferior frontal cortex in one, and the dorsal anterior cingulate cortex (dACC) and posterior cingulate cortex (PCC) in another. Various reference montages and current intensities were also used.

The results of prefrontal stimulation from this review are consistent with research findings that anodal prefrontal tDCS improves executive functions in healthy populations and in those with disorders [49]. However, the effects vary due to various factors, including timing of stimulation, stimulation duration, current intensity, age groups and different cognitive tasks. Future studies should compare the effectiveness of tDCS in improving executive functions in people with HIV, using the same parameters (anodal stimulation of the left DLPFC (F3) with a reference montage on the right supraorbital area (FP2) or right contralateral arm, 2 mA intensity, and a minimum number of sessions of 10). The stimulation of the right inferior frontal cortex was the aim of two studies. This type of stimulation is less frequently tested in the context of improving executive functions. In a study by Jacobson et al. [50], tDCS improved response inhibition in healthy people. Similar results were achieved in a study by Campanella et al. [51]. In another study by Jacobson et al. [52], stimulation of the same region resulted in a significant and selective diminution of the power of theta band in rITG. In another study by Campanella et al. [53], tDCS reduced the amplitude of the P3d component during the response inhibition task. In a study by Sandrini et al. [54] using resting-state functional magnetic resonance imaging in healthy people, tDCS improved response inhibition. Furthermore, during Stop responses, tDCS improved the functional connection between the subthalamic nuclei and the right pre-supplementary motor area (rPreSMA). The utilization of rsfMRI uncovered alterations in the intrinsic connectivity between the right inferior parietal cortex (rIPC), right DLPFC, and the right IFC and caudate. Regarding dACC and PCC stimulation in the study by Jiang et al. [48], their work was the first to test this montage. Further research measuring the mechanisms of this type of stimulation is needed.

### 4.3. Attention

Certain studies [44,45,46] have shown beneficial impacts of tDCS on attention, especially when it comes to tests like Digit Span Backward and Sequencing and Digit Span Forward. Nevertheless, disparities were seen, indicating that the effect of tDCS on attention can vary depending on the activity at hand and may be impacted by personal characteristics. The improvement confirmed by all tests was obtained in studies in which the left DLPFC was stimulated. This montage has proven effective in improving attention in healthy people and people with disorders [55]. In Cody et al. [45], it was observed that tDCS had a positive impact on visual attention. This finding aligns with the results of Gögler et al. [56], who demonstrated that even a single session of excitatory tDCS on the left DLPFC resulted in long-term changes in the frontoparietal alertness networks, leading to improved visual processing speed associated with neurocognitive alertness. These sustained improvements in processing speed could be attributed to the plasticity effects mediated by NMDA receptors induced by tDCS [57,58,59]. Although Gögler et al. [56] focused on patients with major depressive disorder, it is reasonable to assume that similar effects can be observed in other patient populations. The reactivation of the underaroused processing system through tDCS over the DLPFC may lead to increased involvement of neurons in processing visual information and heightened excitability of these neurons. Consequently, enhancing prefrontal activity and modulating functional connectivity in the impaired intrinsic alertness system with tDCS can result in enhanced activation of the visual perceptual system when processing visual target information [56]. tDCS induces changes in neuronal effectiveness and enhances surface sensitivity in the cortex, thereby promoting dopamine release [60,61]. Increased dopamine levels may contribute to improved cognitive abilities [62], and visual attention processes are considered executive and cognitive functions. Consequently, tDCS facilitates learning by modifying neuron sensitivity and displacement potential in the superficial cortex, thereby allowing for increased or decreased brain cell charging. Visual attention processes are associated with brain regions such as the prefrontal cortex, which weak electrical currents can stimulate. This stimulation can either impair or enhance the function of brain neurons [63]. Further exploration is needed to understand the specific role of tDCS stimulation of the right inferior frontal cortex in visual attention processes.

### 4.4. Reaction Time and Psychomotor Speed

Ownby and Kim [43] found conflicting results when examining how tDCS affected psychomotor speed and reaction time. For the WAIS-IV Coding subtest, there was a significant interaction between group and time, indicating enhanced psychomotor speed in the CCT with sham tDCS group, even though tDCS did not improve reaction time or psychomotor speed in general. Using the Grooved Pegboard Test, Cody et al. [45] likewise observed increases in psychomotor speed in the active tDCS group. In contrast to the sham tDCS group, people with HIV in this study showed reduced progress in the Digit Symbol Copy test following SOP training with active tDCS. Therefore, SOP training did not improve psychomotor speed in people with HIV, or learning was slower in these people. Studies have shown that tDCS can reduce cognition-related reaction time in older adults [64]. Various protocols are effective, including stimulation of the left DLPFC. Regarding rIFG stimulation, further research is needed to confirm the effectiveness of this protocol in improving psychomotor speed.

### 4.5. Speed of Processing

Two studies [45,46] showed improvement in SOP in people with HIV, but results were inconsistent related to the variability in the measures used. Speed of processing is hypothesized to be due to specific activation patterns in areas of the prefrontal cortex, specifically the dorsolateral prefrontal cortex [65,66]. It has been suggested that additional prefrontal regions are recruited for those with slower SOP to support successful task performance [65,67]. Some studies suggested that anodal tDCS over DLPFC improves SOP in cognitive tasks in healthy adults and in participants with ADHD [68,69,70]. In studies with HIV patients, the right inferior frontal cortex was stimulated. The role of this area in the SOP remains to be demonstrated in future work.

### 4.6. Depression

tDCS has shown improvements in depression rates among HIV patients. The use of tDCS for depression treatment has been extensively studied, and multiple reviews have highlighted its effectiveness [71,72]. In a study by Brunoni et al. [73], repeated sessions of anodal tDCS targeting the left DLPFC improved the mood of depressed patients, although it did not have a significant impact on blood concentrations of brain-derived neurotrophic factor (BDNF). A study conducted by Jog et al. [74] explored whether repeated tDCS treatments, either with conventional or high-definition setups, could promote structural brain changes in individuals with depression. The findings revealed that tDCS targeting the left DLPFC, as measured by MRI assessments of the magnetic fields induced by the tDCS currents, along with functional modulation of the left DLPFC and the anterior cingulate gyrus, led to alterations in blood flow, as assessed by brain MRI measurements. In the same cohort where the empirical targeting and functional modulation of the left DLPFC had been previously demonstrated with both conventional and high definition tDCS, significant growth of gray matter was observed in the left DLPFC after 12 days of tDCS. Additionally, in the study by Bulubas et al. [75], tDCS combined with MRI was used to investigate whether tDCS induces structural changes in the brain of depressed patients. They found that greater gray matter volumes in the left dorsal prefrontal cortex were associated with improved depression in the tDCS group. Based on the current evidence, it appears that tDCS is effective in treating depression among HIV patients.

### 4.7. Verbal Learning and Memory

In two studies [43,44], stimulation was applied to the DLPFC and showed positive results. These results are consistent with other studies using the same montages [76,77].

### 4.8. Cognitive Functioning

Regarding cognitive functioning, Ownby and Kim [43] suggested enhanced cognitive functioning compared to the control group, with improvements in attention, as seen in the Digit Span Forward subtest. The study by Ownby and Acevedo [44] reported an improvement in the Patient’s Assessment of Own Functioning, reflecting fewer complaints. This coincides with other findings that the tDCS used on the left DLPFC improves cognitive functioning in healthy people and in people with disorders [78,79,80].

### 4.9. The Remaining Studies Showed Null or Mixed Results

Regarding functional abilities, in study [43], there was a moderate effect of tDCS, with improvements observed in certain tasks including the Medication Management Test. On the other hand, there was very little overall impact size for the University of San Diego Scales of Observed Performance. Two studies [43,44] showed improvement in working memory, while one [46] showed no effects. The two studies showing positive results used anodal stimulation on the left DLPFC. A meta-analysis by Mancuso et al. [81] supported these findings, showing that anodal stimulation of the left DLPFC during training led to a small but significant improvement in subsequent working memory performance. Dopamine, a neurotransmitter in the prefrontal cortex, plays a crucial role in working memory [82], and tDCS has been shown to influence dopamine levels in response to stimulation [60,61]. Additionally, the E/I (excitatory–inhibitory) balance theory has been proposed to explain the effects of tDCS on working memory [83]. The E/I balance refers to the relative contributions of excitatory and inhibitory synaptic currents received by a neuron [84]. E/I homeostasis, which maintains a balanced ratio, is a commonly observed phenomenon in the neocortex, including the PFC. The interplay between inhibition and excitation is vital for optimal neural coding and plasticity. Variability in E/I balance is relevant to normal brain function and neuropsychiatric disorders [83]. In HIV-related neurocognitive disorders, excessive activity of extrasynaptic NMDA receptors (eNMDARs) disrupts neural network functioning, leading to compensatory increases in synaptic inhibition [85]. According to Murray and Wang [86], disturbances in the strength of excitatory current conduction on pyramidal cells can disrupt the E/I balance and impair working memory representation. tDCS modulates the E/I balance and working memory by influencing the excitability of interneurons, with the effects influenced by various factors described in detail in the referenced literature [83]. Limited evidence was found regarding the effects of tDCS on sleep, with no significant changes reported in overall sleep quality [45]. According to research, tDCS can improve sleep parameters in healthy people and people with disorders, but stimulation sites other than rIFG have been used so far (e.g., primary motor cortex, M1) [87]. Future studies should investigate the effectiveness of M1 stimulation in improving sleep quality in HIV patients. A study [46] found mixed results regarding language improvement. tDCS has been proven effective in speech rehabilitation in stroke patients [88] and in primary progressive aphasia [89]. Various protocols were used, including the same one as in the study with HIV patients—anodal stimulation of the right inferior frontal gyrus. The lack of results in the HIV sample may be due to the specific pathophysiology of speech disorders occurring in HIV, which is different from that in aphasia. Furthermore, the HIV patients in the study did not suffer from the significant speech impairments that are seen in aphasia. Therefore, due to higher language ability, tDCS may not have produced positive changes that the measurements would have captured. In a study [46], tDCS did not improve episodic memory. Other studies have shown that tDCS improves episodic memory when applied to the prefrontal cortex [90] and temporoparietal cortex [91]. Future studies in HIV patients should test these stimulation areas to investigate the effects of tDCS on episodic memory.

## 5. Limitations and Future Directions

Despite the promising results shown by the studies included in this review, many limitations in these studies make it difficult to interpret and recommend the use of tDCS in patients with HIV.

First, the small sample sizes used in all of the evaluated studies are a noteworthy constraint that could reduce the findings’ statistical power. As an illustration, Ownby and Kim [43] included 46 participants, but Ownby and Acevedo [44] only had 11. Small sample sizes make it difficult to extrapolate the findings to larger populations and raise the possibility of type II errors, which could lead to the misidentification of real effects. Larger and more varied sample sizes should be the goal of future studies to increase statistical robustness and external validity. Second, the studies primarily included elderly adults and people living with HIV, which limited the range of ages, medical problems, and demographic characteristics. This lack of diversity makes it difficult to extrapolate the results to a larger population. Future research should aggressively seek out different participant groups to improve the external validity and generalizability of findings. Including people with varying ranges of age, health issues, and backgrounds will provide information on the possible range of tDCS effectiveness and its suitability for a more diverse population. Third, a recurrent issue concerns the short follow-up durations primarily employed in the investigations. Many evaluations were carried out either right after the intervention or after a short amount of time. This time restriction makes it more difficult to determine the durability and long-term impacts of the cognitive gains brought about by tDCS interventions. Prioritizing the use of rigorous longitudinal study designs in future research will help clarify the long-term impacts of tDCS. Longer follow-up times would offer a more thorough comprehension of the longevity and sustainability of cognitive gains, illuminating the long-term effects of tDCS. Fourth, there may be a confounding factor introduced by the variations in tDCS techniques amongst studies, which include variations in electrode location, current intensity, and session time. tDCS protocols must be standardized and improved to improve study comparability and repeatability. To guarantee uniformity in the delivery of interventions, this entails agreeing on electrode placement, current intensity, and session length. Fifth, some trials did not include a placebo control group. It is noteworthy that Cody et al. [45] did not include a placebo control group for speed of processing training, making it difficult to determine if the effects seen were unique to tDCS or could have been impacted by expectancy or placebo effects. Future research must use strong placebo-controlled procedures. Concerning potential placebo or expectation effects, this strategy guarantees a more accurate evaluation of the specific effects of tDCS. Increasing a study’s internal validity will increase trust in the actual effectiveness of tDCS interventions. Sixth, outcome measures showed heterogeneity. The wide range of outcome measures used in different studies makes it more difficult to synthesize and directly compare findings. A more comprehensive examination of the effects of tDCS interventions would be made easier by standardizing cognitive and functional measures. A consensus on standardized outcome measures should be the goal of future research to facilitate a more thorough understanding of the effects of tDCS across many cognitive areas. Seventh, it is important to look at the effects of different intervention combinations. Studies that combined tDCS with another intervention were examined in a few of the included studies. Subsequent investigations ought to examine whether these amalgamated methodologies yield more intricate and efficacious results in the augmentation of cognition, thereby potentially opening the door for all-encompassing and cohesive intervention tactics. Future research should establish comparison groups between tDCS by itself and tDCS in conjunction with an intervention. Eighth, it is important to control for factors affecting the development of cognitive disorders in people with HIV in future research. It has been shown that the development of cognitive deficits in people from HIV is influenced by factors such as age [92], nadir CD4 count [93], coexistent cardiovascular disease and obesity [93], coinfection with hepatitis C [93], history of toxoplasmosis [93], drug use [94], genetic factors [95], reduced educational attainment [96,97], early immunosuppression [96,97], and elevated MCP-1 and TNF-α plasma concentrations [96,97]. It is worth controlling whether, and if so, how, these factors can affect the results of the treatment of cognitive deficits in HIV in the tested samples. Ninth, in future studies, neuroimaging methods (e.g., fMRS, EEG) and biomarkers of cognitive functions in HIV should be included to better understand the mechanisms of tDCS. The following works can help in the search of useful biomarkers [98,99].

## 6. Conclusions

To summarize, studies analyzing the use of tDCS in individuals with HIV revealed considerable variability in terms of protocols and outcomes. Nonetheless, the available evidence suggests that tDCS can be a beneficial and safe method for enhancing cognitive performance and depression in this population. However, there is still limited knowledge regarding the underlying mechanisms of tDCS and the optimal protocols to achieve the desired effects. To gain a deeper understanding of how tDCS affects the brains of individuals with HIV, it is crucial to conduct new randomized controlled trials that incorporate neuroimaging studies and biomarkers.

## Figures and Tables

**Table 1:** Summary of main findings of articles included in this review.

Author, Citation	Population	Test Used	Intervention	Stimulation Site	Current Intensity	Duration (min)	Main Findings in Treatment Group
Ownby and Kim [43]	46 participants and 3 groups: (1) cognitive training with active tDCS (n = 16), (2) cognitive training with sham tDCS (n = 15), (3) watching educational videos with sham tDCS (n = 15)	CCAP, the Stroop Color Word Test, the Iowa Gambling Task, the Design Fluency subtest of the Delis-Kaplan Executive Function System, MMT, UPSA, HVLT-R, the Digit Span subtests from the WAIS-IV, PAOF	6 sessions	Anode placed over the left DLPFC, F3 in 10-20 System, and cathode over the right supraorbital area (FP2)	1.5 mA	20	- the Trail Making Test Part B did not show significant group and time interaction effects, but the results for the active treatment group nearly reached significance at the second follow-up. For the Stroop test, significant improvements were noted in the cognitive training group with sham stimulation, showing a large effect size, while other tests showed non-significant moderate improvements; - cognitive training, with or without tDCS, improved cognitive functions compared to the control group. The Digit Span Forward test almost reached statistical significance, suggesting notable improvements. Other attention tests had positive yet non-significant outcomes. All groups improved similarly across assessments in PAOF outcomes; - tDCS did not enhance reaction time or psychomotor speed. Trails B test results were close to significant when comparing the active treatment group with the control group, becoming significant at the second follow-up. Significant improvements were observed in the WAIS-IV Coding subtest primarily due to the CCT with sham stimulation group’s performance; - the MMT showed a moderate but non-significant effect size, while the UPSA showed a negligible effect; - the HVLT-R indicated significant improvements in both treatment groups over time, with notable differences between the CCT with active tDCS group and the control group immediately after the treatment, which decreased over time; - the Digit Span Forward subtest showed improvements in working memory; - depression levels remained similar across all groups, except for improved mood in the active treatment group one month after treatment
Ownby and Acevedo [44]	11 participants: active tDCS = 6, sham tDCS = 5	The WAIS-IV with Digit Span subtest—tests of digit span forward and backwards, number and letter sequencing task, the Trail Making Test, Parts A and B, the HVLT-R, the Grooved Pegboard Test, CESD, PAOF	6 sessions	Anodal tDCS over the left DLPFC, cathode placed over the right supraorbital area	1.5 mA	20	- there was an improvement in executive functions measured using the Trail Making Test, Parts A and B; - improvement in PAOF after tDCS (fewer complaints); - results for the Grooved Pegboard dominant hand time showed improvement in the active tDCS group; - improvement in HVLT-R in both groups, but in active tDCS group the improvement was greater; - improvement in working memory, specifically in tasks such as Digit Span Backward and Sequencing; - findings showed improvement in attention in tasks such as Digit Span Backward and Sequencing; - reduced CESD depression scores
Cody et al. [45]	66 participants in 4 groups: (a) HIV-positive with tDCS (n = 17), (b) HIV-positive with sham tDCS (n = 16), (c) HIV-negative with tDCS (n = 17), and (d) HIV-negative with sham tDCS (n = 16)	The WAIS Digit Symbol Substitution Test, Digit Symbol Copy test, UFOV, PSQI, Letter and Pattern Comparison Test	10 sessions	The anodal electrode placed over the right inferior frontal cortex near F10, while the cathode electrode was placed on the contralateral upper arm	2.0 mA	20	- people with HIV achieved less improvement in the Digit Symbol Copy test after SOP training with active tDCS compared to people after sham tDCS; - in the Digit Symbol Substitution Test, there was no improvement after active tDCS. All groups improved on the UFOV; - the HIV-positive group that received tDCS with SOP training showed significant improvements in the Letter Comparison Test. The groups did not show any notable variations in their Pattern Comparison scores; - no significant changes were seen in the groups’ overall quality of sleep (all *p* values > 0.05)
Fazeli et al. [46]	33 participants: active tDCS (n = 17), sham tDCS (n = 16)	NIHTB-CB with tests Flanker and Dimensional Card Change Sorting, Picture Sequence Memory Test, Picture Vocabulary Test, Oral Reading Recognition Test, Letter and Pattern Comparison Test, Digit Symbol Substitution Task, List Sorting Test, UFOV	10 sessions	The F10 electrode was used as the anode (right inferior frontal cortex), and the cathode electrode was placed on the contralateral upper arm	2.0 mA	20	- there was improvement in executive functions measured by Dimensional Card Change Sorting and Flanker; - there was no improvement in working memory as measured by the List Sorting Test; - no improvement in UFOV subtest 2 (divided attention). The outcomes of subtest 3 (selective attention) improved significantly in the tDCS group at a significance level of *p* < 0.05 after applying correction using Holm’s procedure; - an improvement was shown in speed of processing measured by the Letter and Pattern Comparison Test. The outcomes of the Digit Symbol Substitution Task improved significantly in the tDCS group at a significance level of *p* < 0.05 after applying correction using Holm’s procedure; - language improvement was demonstrated as measured by the Oral Reading Recognition Test. Conversely, there was no evidence of language improvement as measured by the Picture Vocabulary Test; - no improvement in episodic memory as measured by the Picture Sequence Memory Test
Knotkova et al. [47]	10 participants: only active tDCS (n = 10)	HDRS, MADRAS, and MMSE were used	10 sessions	The anode was placed over the F3 (left DLPFC) position according to the EEG 10-20 system, while the cathode was positioned over the contralateral supraorbital region	2.0 mA	20	- the results showed a significant decrease in depression scores (*p* < 0.0005) after the tDCS treatment. HDRS scores for the eight patients averaged 26.3 (s.d. = 5.5) at baseline, 9.9 (s.d. = 4.3) immediately after the last tDCS session, and 7.6 (s.d. = 6.7) at the two-week follow-up. Similarly, MADRAS scores averaged 26.8 (s.d. = 6.7) at baseline, 11.3 (s.d. = 6.9) immediately after the last tDCS session, and 7.0 (s.d. = 7.4) at the two-week follow-up. MMSE scores remained stable or improved after the treatment
Jiang et al. [48]	11 participants: active tDCS (n = 7), sham (n = 4)	TMT-A, TMT-B, SCWT, WCST	10 sessions	Finite element electrical field models guided the placement of two anodal electrodes at AFz and CPz, with two cathodal return electrodes at T7 and T8, respectively	1.5 mA	20	- real tDCS when compared to sham tDCS resulted in a decrease in Perseverative Errors during the WCST but did not have an effect on Non-Perseverative Errors. Additionally, there was a decrease in the ratio score of TMT-B to TMT-A). There was no effect on SCWT

Abbreviations: DLPFC—dorsolateral prefrontal cortex, PAOF—Patient’s Assessment of Own Functioning, MMT—Medication Management Test—Revised, UPSA—University of San Diego Scales of Observed Performance, CESD—Center for Epidemiological Studies Depression scale, CCAP—California Computerized Assessment Package, HVLT—R-Hopkins Verbal Learning Test—Revised, WAIS-IV—Wechsler Adult Intelligence Scale, 4th edition, UFOV—Useful Field of View, PSQI—Pittsburgh Sleep Quality Index, NIHTB-CB—NIH Toolbox Cognition Battery, HDRS—Hamilton Depression Rating Scale, MADRAS—Montgomery–Asberg Depression Rating Scale, MMSE-Mini—Mental State Examination, WCST—Wisconsin Card Sorting Test, TMT-A—Trail Making Test-Part A, TMT-B—Trail Making Test-Part B, SCWT—Stroop Color and Word Test.

## Data Availability

No new data were created or analyzed in this study. Data sharing is not applicable to this article.
